# Bedaquiline: A Novel Diarylquinoline for Multidrug-Resistant Pulmonary Tuberculosis

**DOI:** 10.7759/cureus.28519

**Published:** 2022-08-29

**Authors:** Anuradha T Deshkar, Prashant A Shirure

**Affiliations:** 1 Department of Pharmacology, Datta Meghe Institute of Medical Sciences, Nagpur, IND; 2 Department of Pharmacology, Dr. Vaishampayan Memorial Government Medical College, Solapur, IND

**Keywords:** heteroresistance, culture conversion rate, pulmonary tuberculosis, drug resistance, multidrug-resistant tuberculosis, diarylquinoline, bedaquiline

## Abstract

A new drug Bedaquiline, a diarylquinoline agent has been approved by the Food and Drug Administration for the treatment of pulmonary multidrug-resistant tuberculosis. It has been given approval for use along with the basic regimen with only conditional access through the National Program for tuberculosis in India. The major problem with existing antitubercular drugs used for the treatment of multi-drug resistant tuberculosis is antimicrobial resistance, less efficacy, and poor side effect profile. Bedaquiline might be a solution to these issues. Bedaquiline is a first of its class drug with a unique and specific mechanism of action. It inhibits mycobacterial adenosine triphosphate (ATP) synthase's proton pump. There are many randomized clinical trials and cohort studies that reported a higher culture conversion rate with bedaquiline treatment as compared to the control group. Many meta-analyses and systematic reviews have reported higher culture conversion rate, higher cure rate, and lower mortality rate in patients with drug-resistant tuberculosis treated with a bedaquiline-containing regimen. Here is a detailed drug profile of bedaquiline to help health care workers treat tuberculosis patients.

Keywords:

## Introduction and background

Tuberculosis (TB) is caused by Mycobacterium tuberculosis [1]. This contagious infection contributes to a major cause of illness and deaths occurring in India [2]. India has the highest global burden of TB which accounts for almost a quarter of cases of TB from all over the world [3]. The occurrence of drug-resistant strains is one of the major issues with pharmacotherapy of this disease [4,5]. Resistance to only Rifampicin is known as rifampicin-resistant TB (RR-TB). Multidrug-resistant TB (MDR-TB) is characterized by resistance to rifampicin (R) as well as isoniazid (H) with or without resistance to first-line antitubercular drugs [4]. Resistance to rifampicin, isoniazid, and any of the fluoroquinolone (FQ) along with at least any one of the group A drugs is known as extensively drug-resistant TB (XDR-TB), whereas resistance to all medications is known as totally drug-resistant TB (TDR-TB) [4]. It takes years and costs a lot to treat drug-resistant TB (DRTB) as compared to treating ordinary diseases [5]. As a result, novel anti-tubercular medicines with a distinct mode of action are urgently needed [1,4]. Rifapentine, an antitubercular drug was approved in 1998. After this, no other antitubercular drug was approved for a long duration, and then bedaquiline, a novel antitubercular drug was approved in 2012. There are numerous medications under phase II as well as phase III trials for better management of TB. Bedaquiline is one such drug that is first in class and can help in the better management of TB [6]. It was discovered by scientists by Johnson & Johnson at their Janssen pharmaceutical division [5]. This medicine has received accelerated approval from the United State Food and Drug Administration (USFDA) [7]. Initially, this drug had been available to individual patients under “compassionate use” with pre-approval of the Drug Controller General of India ( DCGI) upon request of the treating physician, who submits patients' details fulfilling the USFDA criteria for accessing the drug from Janson as a donation [4]. Now, the National Tuberculosis Elimination Program (NTEP) with Programmatic Management of Drug-Resistant TB (PMDT) services has approved bedaquiline-containing regimens for the treatment of DRTB and bedaquiline has been rolled out for all DRTB centers in India [4,8]. Every registered medical practitioner in India must know about bedaquiline in detail which is an important drug in the treatment of DRTB [6]. Here we are providing a drug profile of bedaquiline that will be useful to health care workers in India while prescribing bedaquiline for the treatment of DRTB.

## Review

Chemistry

Bedaquiline is a diarylquinoline having alcohol and amine groups on its side chains [9-11]. its antimycobacterial effect is due to these two side chains. It has a quinolinic central heterocyclic nucleus [9]. Molecular formula: C33H31BrN2O2. Molecular weight: 555.504 g/mol. ATC Code: J04AK05 - Bedaquiline [10]. Fumaric acid in ratio 1:1 is used with bedaquiline to prepare bedaquiline fumarate [12]. In enantiopure bedaquiline, there are two chiral centers in it. There is a mixture of four isomers. Bedaquiline is isolated from it. It's the most effective stereoisomer against several mycobacterial strains [13]. The configuration of bedaquiline is identified through conformational analysis along with x-ray diffraction research [12,14].

Mechanism of action 

Bedaquiline possesses antimycobacterial activity that is both distinct and specific. It inhibits mycobacterial adenosine triphosphate (ATP) synthase's proton pump. Prokaryotic as well as eukaryotic cells require the production of ATP for cell life. It is done with the help of ATP synthase. Bedaquiline inhibits the generation of ATP. It binds to mycobacterial ATP synthase at subunit c which is oligomeric and proteolipid. As a result, it causes bacterial death [9,15]. Bedaquiline binds to mycobacterial ATP synthase with more than 20,000 times more affinity than it binds to human mitochondrial ATP synthase. It is the reason for specific action in mycobacterium only and minimum host cell damage [16].

 Antimicrobial spectrum

By blocking mycobacterial ATP synthase, bedaquiline kills dormant as well as actively reproducing mycobacteria. It inhibits drug-resistant mycobacterium along with drug-sensitive mycobacterium [17,18]. Bedaquiline has a substantial inhibitory impact on a wide range of nontuberculous mycobacteria (NTM), including Mycobacterium avium, Mycobacterium ulcerans, Mycobacterium abscessus, and Mycobacterium intracellulare [18]. Bedaquiline has a mild inhibitory effect on Gram-positive bacteria and Gram-negative bacteria [18,19].

Drug resistance

Bedaquiline-resistant mutations are found in one out of every 10^8 ^organisms [18]. There are two mechanisms for the occurrence of mutation for bedaquiline. One is the atpE gene mutation and the other is the expression of the efflux pump. Mutations in the atpE gene at position 63 and position 66 affect bedaquiline's ability to bind to ATP synthase enzyme's c subunits [9,20]. The fast-growing Mycobacterium novocastrense along with the slow-growing NTMs Mycobacterium shimoidei and Mycobacterium xenopi, both have atpE gene variations. Bedaquiline resistance comes easily due to this [19-21]. Expression of efflux pump leads to drug efflux enhancement which is the mechanism behind bedaquiline resistance. The expression of the efflux pump is induced by mutations in Rv0678 [22]. Mutation in Rv0678 is also responsible for heteroresistance in M. tuberculosis for bedaquiline. Heteroresistance is the phenomenon where bacterial isolates contain subpopulations with increased antibiotic resistance along with antibiotic-susceptible populations [23]. No cross-resistance is observed with other antitubercular medications (rifampin, fluoroquinolones, PA-824, amikacin, ethambutol, moxifloxacin, isoniazid, streptomycin, pyrazinamide) [24].

Pharmacokinetics

Absorption

Bedaquiline is well absorbed in humans after single and several doses of oral administration. Regardless of the dose, it achieves its maximum plasma concentration in 4-6 hours after delivery [25]. With increasing dose, the area under the curve (AUC) and the Cmax increase proportionally. Up to levels of 700 mg, it has a linear pharmacokinetic profile [18]. Bedaquiline has concentration-dependent bactericidal activity. The area under the curve (AUC) determines its activity [26]. Steady-state plasma concentration is 600 ng/mL and does not reach even after seven days. Administration with a meal doubles its bioavailability as compared to its administration on an empty stomach [24,27].

Distribution

Bedaquiline has a plasma protein binding rate of >99.9% [18]. The central compartment of bedaquiline has a distribution volume of 164 L [26]. 5 mg/L is Cmax of bedaquiline in sputum after administration of 400 mg once daily for seven days. The comparable Cmax is obtained in plasma after administration of the same dose in a treatment-naive patient. As no clinical trial has studied the distribution of bedaquiline in other tissues data regarding this is not available [25]. Bedaquiline has a half-life of 164 days while that of M2 is 159 days. Cationic amphiphilic properties are present in each of these molecules. As a result, these chemicals are released slowly from peripheral tissues, resulting in a prolonged half-life [25,28]. Bedaquiline and M2, accumulate in tissues, due to their binding to intracellular phospholipids [25].

Metabolism

Bedaquiline undergoes phase I metabolism in humans. It undergoes N-methylation in the liver. It is processed by CYP3A4, CYP2C8, and CYP2C19. A major role is of CYP3A4. The final product is N-monodesmethyl metabolite, M2 [24,25,27]. The potency of M2 is lower than the parent drug. Its average exposure is also low as compared to parent drugs [29]. Another, quantitatively less important metabolite with a negligible antimycobacterial activity that is formed by N-demethylation of M2 is N-desmethyl metabolite, M3. Possession of greater cytotoxicity and phospholipidogenic potential by both M2 and M3 compared to bedaquiline is shown in in-vitro studies [25].

Excretion

Bedaquiline is primarily eliminated by feces [25,28]. In feces, 75-85% is eliminated in unchanged form and 3.7-7.2% is M2. Elimination of unchanged form in the urine is less than 0.001% of the administered dose. Bedaquiline has a half-life of approximately 24 hours [28].

Indications

The only indication is the treatment of pulmonary TB due to MDR M. tuberculosis as part of combination therapy in adults (≥ 18 years) [24].

Dose, dosage form, and strength

For the first two weeks, 400 mg (four tablets of 100 mg) of bedaquiline is prescribed which is needed to be taken once daily. From weeks 3 to 24, 200 mg (two tablets of 100 mg) of bedaquiline need to be administered three times per week with at least 48 hours between two consecutive doses [30]. Bedaquiline is available in tablet form with the strength of 100 mg [30].

Adverse effects

Nausea, vomiting, diarrhea, pain abdomen, limb pain, arthralgia, back pain, headache, and dizziness are some of the adverse drug reactions observed with the use of bedaquiline related to the gastrointestinal system, and musculoskeletal system, and central nervous system. Rash, pruritus, acne, hemoptysis, pleuritic pain, pharyngolaryngeal pain, deafness, hyperuricemia, QT interval prolongation, and elevated transaminases are some other reported adverse effects of bedaquiline administration [9,29-32].

Drug-drug interactions

Antitubercular Medications

Coadministration of bedaquiline and rifampicin showed a major (52%) reduction of bedaquiline exposure, which results in ineffective potential. So bedaquiline should be avoided with a strong CYP3A4 inducer [24]. Other antitubercular drugs: There are no significant pharmacokinetic interactions with like ethambutol, kanamycin, isoniazid, Pyrazinamide, ofloxacin, or cycloserine [24,27].

Antiretroviral Medications

Lopinavir acts as both substrate and an inhibitor of CYP3A4, so the combination of lopinavir and ritonavir increases the bioavailability of bedaquiline by 22% without affecting Cmax [25,30]. With nevirapine, bedaquiline can be safely administered without dosage alteration [29]. Because of metabolism by CYP2B6 and CYP3A and induction of CYP3A, the chronic coadministration of efavirenz can limit exposure to bedaquiline and its major metabolite M2 by approximately 50% [25]. Coadministration of bedaquiline with ketoconazole results in an increase in AUC (strong CYP3A4 inhibitors) [30]. Prescribing bedaquiline along with the drugs causing Prolonged QT intervals like ketoconazole, fluoroquinolones, macrolide, and clofazimine resulted in additive or synergistic QT prolongation [33].

Special population

Special Physiological States

Bedaquiline comes under Category B in the classification of drugs used in pregnancy [30]. The data on whether bedaquiline is secreted in breast milk is not studied in any clinical trials; so need to be used with caution in nursing mother [29]. Clinical data on the pediatric and geriatric population to establish safety and efficacy are not available [30].

Special Disease Conditions

No need for alteration of dose in mild and moderate hepatic failure. In patients with severe hepatic impairment, caution needs to be taken while administrating bedaquiline [29]. No need for alteration of dose in mild to moderate renal failure. In patients with severe renal impairment and those who are on dialysis, caution need to be taken while administrating bedaquiline [29]. The complete drug profile of bedaquiline is tabulated in Table 1.

**Table 1:** A complete drug profile of bedaquiline

Sr. No	Characteristics	Information of Bedaquiline
1	Chemistry	A diarylquinoline having alcohol and amine groups on its side chains
2	Molecular formula	C33H31BrN2O2
3	Molecular weight	555.504 g/mol
4	ATC Code	J04AK05 – Bedaquiline
5	Mechanism of action	Inhibits mycobacterial adenosine triphosphate (ATP) synthase's proton pump
6	Antimicrobial spectrum	Dormant as well as actively reproducing mycobacteria, drug-resistant mycobacterium, nontuberculous mycobacteria, gram-positive bacteria and gram-negative bacteria
7	Drug resistance mechanisms	atpE Gene Mutation and Expression of Efflux Pump
8	Absorption	well absorbed in humans orally
9	Volume of Distribution	164L
10	Half-life	164 Days
11	Metabolism	N-methylation by by CYP3A4, CYP2C8 and CYP2C19 in liver
12	Excretion	75-85% in faeces
13	Indications	Pulmonary tuberculosis due to MDR M. tuberculosis as part of combination therapy in adults (≥ 18 years)
14	Dose	Weeks 1-2: 400 mg (4 tablets of 100 mg) once daily Weeks 3-24: 200 mg (2 tablets of 100 mg) 3 times per week
15	Drug-drug interactions	With some antitubercular drugs, antiretroviral and antifungal drugs

Real-world evidence

Under NTEP, India has introduced three main bedaquiline-containing regimens for the treatment of DRTB [4]. The first one is a shorter oral bedaquiline-containing MDR/RR-TB regimen. It is indicated for the treatment of adults suffering from MDR-TB and RR-TB and those who have not been exposed to second-line antitubercular drugs, used in this regimen, for more than one month. Patients with isoniazid and fluoroquinolone resistance have been excluded from this regimen. This regimen contains an initial phase (IP) of four months which can be extended up to six months and a continuation phase (CP) of five months. So, the total duration of the regimen is nine to 11 months. The IP contains seven drugs bedaquiline (Bdq 400 mg to be taken once daily for the first two weeks, from weeks 3 to 24, 200 mg three times a day), levofloxacin (Lfx), clofazimine (Cfz), pyrazinamide (Z), ethambutol (E), high dose isoniazid (H^h^), and ethionamide (Eto). The continuation phase contains four drugs levofloxacin, clofazimine, pyrazinamide, and ethambutol. Bedaquiline is given for six months. For the first four months, all seven drugs are given Bdq, Lfx, Cfz, Z, E, H^h^, and Eto. For the fifth and sixth months, five drugs are given, if IP is not extended and those are Bdq, Lfx, Cfz, Z, and E. From the seventh month to the 11th month four drugs Lfx, Cfz, Z, and E are given. If IP is extended, then all three drugs Bdq, H^h,^ and Eto are stopped together at the end of six months. Programmatic data from South Africa which was reviewed by World Health Organization (WHO) reported that there is a 13% increase in success rate with a shorter bedaquiline-containing regimen compared to a shorter injectable-containing regimen [4].

Another bedaquiline-containing regimen is a longer oral M/XDR-TB regimen for RR-TB, MDR-TB and XDR-TB [4]. This regimen is indicated for patients suffering from MDR-TB and RR-TB but excluded from a shorter oral bedaquiline-containing MDR/RR-TB regimen and for patients suffering from extensively DRTB. This regimen is for 18-20 weeks without any IP and CP. WHO recommends starting with all three groups A drug and at least one group B drug and at least three drugs should be continued if Bdq is stopped. In India, experts concurred to start with all five group A and group B drugs and continue with four drugs in the later part of treatment. It contains five drugs Lfx, Bdq, linezolid (Lzd), Cfz, and Cs in starting months. At the end of six months regimen is modified depending on the culture report of month 5. The duration of bedaquiline in this regimen is only six months. If it is not possible to design an alternative regimen then only bedaquiline can be administered up to a maximum extended period of 20 weeks. Although standardization of the regimen could be done modifications in composition and duration according to individual patients make the regimen more effective and safer. Programmatic data from South Africa which was reviewed by World Health Organization (WHO) reported that there is a 13% increase in success rate with a longer oral M/XDR-TB regimen compared to a shorter injectable-containing regimen [4].

Bedaquiline, Pritomanide, and Linezolid (BPaL) regimens are new regimens suggested by WHO. It is included in the guidelines of NTEP for the treatment of multidrug resistance TB with additional fluoroquinolone resistance. This regimen is for 26 weeks which can be extended up to 39 weeks if the patient is culture-positive at 16 weeks. BPaL showed 90% favorable outcomes among XDR (89%), MDR with FQ resistance, and treatment intolerant/non-responders (92%) [4].

In a randomized control trial, conducted on 160 patients, bedaquiline and placebo were added to the preferred background regimen. This study reported that median culture conversion time in the bedaquiline treated group was reduced to 83 days from 125 days (95% confidence interval, p<0.001 by Cox regression analysis) [34]. In a study conducted in South Africa, 272 newly diagnosed patients with XDR-TB were prospectively observed from 2008 to 2017. This study reported that in the bedaquiline group the favorable outcome rate was better than in the nonbedaquiline group (66.2% versus 13.2%; p<0.001) [35]. A systematic review and analysis were carried out in 2021 which included a total of eight studies. Out of a total of eight studies, two were randomized control trials and the remaining six were cohort studies. This study reported that there was a higher culture conversion rate in the bedaquiline group compared to the control group (risk ratio [RR]: 1.272, p<0.001) [36]. In a meta-analysis, 50 studies were considered from 25 different countries, and data from 12,030 patients were studied. This study reported treatment success was positively associated with bedaquiline (adjusted risk difference 0.10, 95% confidence interval) [37]. A systematic review and meta-analysis published in 2022, considered 25 observational studies and 4 experimental studies where MDR-TB patients were treated with the bedaquiline-containing regimen. This study reported a treatment success rate of 74.4% in observational studies and 86.1% in experimental studies with bedaquiline treatment [38]. A study evaluated the pharmacokinetic-pharmacodynamic relationship between different antitubercular drugs and their use while selecting drug combinations for DRTB. This study reported that pyrazinamide increases the potency of bedaquilie by two folds [39]. An individual patient data meta-analysis studied data of 11,920 DRTB patients and reported that the use of any group A antitubercular drug such as bedaquiline is associated with significantly decreased odds of death [40]. A cross-sectional cohort study reported that bedaquiline containing regimen used in DRTB needs a functional background regimen to achieve a high cure rate and prevent evaluation of bedaquiline resistance. This study also reported that novel bedaquiline regimens require timely and comprehensive drug-resistance monitoring [23]. A cross-sectional analysis done in 2023 patients and a longitudinal analysis done in 695 patients reported that bedaquiline resistance is associated with poorer treatment outcomes. If the patient is previously exposed to bedaquiline or the patient remains culture positive after two months of treatment, rapid assessment of bedaquiline resistance should be prioritized [41]. A study was done on 177 MDR-TB and XDR-TB patients in China and reported that bedaquiline, when added to the background regimen, is associated with a high rate of culture conversion. Culture conversion rate was 84.6% for MDR-TB, and 86.6% for XDR-TB with 95% confidence interval [42].

## Conclusions

India is the capital of TB. The emergence of DRTB is a vital issue in the treatment of TB. Bedaquiline has now been incorporated into the National Tuberculosis Elimination Program for the pharmacotherapy of DRTB. Many systematic reviews and meta-analyses performed all over the world have reported better culture conversion rates, increased cure rates, and decreased mortality rates with the treatment of bedaquiline. Each registered medical practitioner in India should have at least basic knowledge about the drug profile of bedaquiline to prescribe and monitor the treatment of DRTB confidently and rationally. Rational use of bedaquiline will enhance the efficacy of pharmacotherapeutic management of DRTB and decrease the side effects of the drug and also help in decreasing the chances of resistance.
